# Comparison of the Diagnostic Accuracy of Three Rapid Tests for the Serodiagnosis of Hepatic Cystic Echinococcosis in Humans

**DOI:** 10.1371/journal.pntd.0004444

**Published:** 2016-02-12

**Authors:** Francesca Tamarozzi, Ilaria Covini, Mara Mariconti, Roberta Narra, Carmine Tinelli, Annalisa De Silvestri, Federica Manzoni, Adriano Casulli, Akira Ito, Andreas Neumayr, Enrico Brunetti

**Affiliations:** 1 Department of Clinical Surgical Diagnostic and Paediatric Sciences, University of Pavia, Pavia, Italy; 2 WHO Collaborating Centre for the Clinical Management of Cystic Echinococcosis, Pavia, Italy; 3 Clinical Epidemiology and Biometry Unit, San Matteo Hospital Foundation, Pavia, Italy; 4 Department of Infectious, Parasitic and Immunomediated Diseases, Istituto Superiore di Sanitá, Roma, Italy; 5 Department of Parasitology, Asahikawa Medical University, Asahikawa, Japan; 6 Medical Services and Diagnostic, Swiss Tropical and Public Health Institute, and University of Basel, Basel, Switzerland; 7 Division of Tropical and Infectious Diseases, San Matteo Hospital Foundation, Pavia, Italy; Institute of Tropical Medicine (NEKKEN), JAPAN

## Abstract

**Background:**

The diagnosis of cystic echinococcosis (CE) is based primarily on imaging, in particular with ultrasound for abdominal CE, complemented by serology when imaging results are unclear. In rural endemic areas, where expertise in ultrasound may be scant and conventional serology techniques are unavailable due to lack of laboratory equipment, Rapid Diagnostic Tests (RDTs) are appealing.

**Methodology/Principal Findings:**

We evaluated the diagnostic accuracy of 3 commercial RDTs for the diagnosis of hepatic CE. Sera from 59 patients with single hepatic CE cysts in well-defined ultrasound stages (gold standard) and 25 patients with non-parasitic cysts were analyzed by RDTs VIRapid HYDATIDOSIS (Vircell, Spain), Echinococcus DIGFA (Unibiotest, China), ADAMU-CE (ICST, Japan), and by RIDASCREEN Echinococcus IgG ELISA (R-Biopharm, Germany). Sensitivity, specificity and ROC curves were compared with McNemar and t-test. For VIRapid and DIGFA, correlation between semiquantitative results and ELISA OD values were evaluated by Spearman’s coefficient. Reproducibility was assessed on 16 randomly selected sera with Cohen’s Kappa coefficient. Sensitivity and Specificity of VIRapid (74%, 96%) and ADAMU-CE (57%, 100%) did not differ from ELISA (69%, 96%) while DIGFA (72%, 72%) did (p = 0.045). ADAMU-CE was significantly less sensitive in the diagnosis of active cysts (p = 0.019) while DIGFA was significantly less specific (p = 0.014) compared to ELISA. All tests were poorly sensitive in diagnosing inactive cysts (33.3% ELISA and ADAMU-CE, 42.8% DIGFA, 47.6% VIRapid). The reproducibility of all RDTs was good-very good. Band intensity of VIRapid and DIGFA correlated with ELISA OD values (r = 0.76 and r = 0.79 respectively, p<0.001).

**Conclusions/Significance:**

RDTs may be useful in resource-poor settings to complement ultrasound diagnosis of CE in uncertain cases. VIRapid test appears to perform best among the examined kits, but all tests are poorly sensitive in the presence of inactive cysts, which may pose problems with accurate diagnosis.

## Author Summary

Cystic echinococcosis (CE) is a parasitic zoonosis prevalent worldwide, especially in economically poor livestock raising areas. Parasitic cysts develop most commonly in the liver and are diagnosed primarily by ultrasound. Serology helps with diagnosis, particularly when ultrasound features are unclear. Unfortunately, in underserved endemic rural areas, expertise in ultrasound diagnosis of CE may be scant, and conventional serology techniques are unavailable due to the lack of laboratory equipment. In these circumstances, Rapid Diagnostic Tests (RDTs) may be very useful. In this work, we evaluated the diagnostic performance of three RDTs and compared them with a commercial ELISA test routinely used in our diagnostic laboratory. Our results show that RDTs have overall comparable performances to ELISA in the diagnosis of hepatic CE in well-defined stages, although significant differences exist among them. If confirmed and expanded on a bigger cohort, these results would support the use of RDTs instead of conventional techniques to complement imaging in the diagnosis of CE.

## Introduction

Cystic echinococcosis (CE) is a parasitic zoonosis caused by the larval stage of the dog tapeworm *Echinococcus granulosus* complex. The parasite is transmitted between canids (definitive hosts harboring in the intestine the adult stage of the tapeworm), and livestock, particularly sheep (intermediate hosts becoming infected by fecal-oral route with eggs shed with dog feces). In the intermediate host, the larval stage develops as an expanding fluid-filled cyst, which can infect the definitive host eating infected organs. Humans behave as accidental intermediate hosts, where CE cysts develop mostly in the liver, followed by lungs. The infection is prevalent worldwide especially in rural livestock-raising areas such as the Mediterranean, Eastern Europe, North and East Africa, South America, Central Asia, China and Australia. The most recent estimates indicate 1.2 million people affected worldwide with 3.6 million Disability Adjusted Life Years lost due to human disease and over 2,190 million USD lost yearly in animal production [1].

Human CE is a chronic, clinically complex and neglected disease [2]. The spectrum of clinical manifestations range from asymptomatic to serious, even life-threatening conditions. Most cases remain a- or pauci-symptomatic for years or even decades and maybe diagnosed accidentally. The diagnosis of human CE is mainly based on imaging. Ultrasound (US) is the imaging technique of choice for the diagnosis of abdominal CE [3]. The current international WHO-IWGE (Informal Working Group on Echinococcosis) classification of CE cyst stages is based on the pathognomonic features of cysts on US, and guides their clinical management [4, 5].

Serology should complement imaging-based diagnosis when imaging features are unclear, although currently available serology tests are burdened by the lack of standardization and by unsatisfactory sensitivity and specificity [6, 7].

In underserved rural endemic areas, the diagnosis of CE poses important problems as expertise in US diagnosis and management of CE may be scant and/or difficult to access, and conventional serology techniques are unavailable or unreliable due to the lack of laboratory equipment. These conditions may not only cause under-diagnosis of CE in patients requiring therapy, but also result in poor differential diagnosis and unnecessary or inappropriate treatments. This is particularly true when serology is used alone without visualization of a compatible lesion by imaging, as the positive predictive value of CE serodiagnosis is very low [8], and when lesions do not show pathognomonic signs of a parasitic origin, such as young CE1 cysts or inactive CE4–CE5 cysts. Unfortunately, these stages are also those with the broader differential diagnosis (e.g. simple cysts, neoplastic lesions) whose serology results are also difficult to interpret and often negative [9].

The use of Rapid Diagnostic Tests (RDTs) is particularly useful is resource-poor settings, and in the context of CE they may be suitable to complement imaging where diagnosis is uncertain. Several reports described the performance of commercial and experimental RTDs in the diagnosis of CE [10–15]; however, no study so far compared the performance of commercially available RDTs. Here we performed a comparison of the diagnostic performance and reproducibility of three commercially available RDTs for the diagnosis of CE and compared them with those of a commercial ELISA test routinely used in the parasitology diagnostic laboratory of San Matteo Hospital Foundation, Pavia, Italy. Our results show that the evaluated RDTs have an overall comparable performance to the ELISA test in the diagnosis of hepatic CE in well-defined stages, although significant differences exist among them. If confirmed in a bigger cohort, these results would support the use of RDTs instead of conventional techniques to complement imaging in the diagnosis of CE.

## Methods

### Patients and sera

Sera included in the analysis were frozen (-80°C) stored samples from patients with hepatic CE and non-parasitic hepatic cysts seen between 2010 and 2015 in the Ultrasound Diagnostic Service of the Division of Infectious and Tropical Diseases, San Matteo Hospital Foundation, Pavia, Italy, where the WHO Collaborating Centre for Clinical Management of Cystic Echinococcosis is based. Clinical information related to each patient and sample was retrieved retrospectively in March 2015 from the electronic database of patients visited in the Centre. Patients included in the study formed a convenience series. Selection criteria were presence of a single cyst, located in the liver, of non-parasitic nature (controls) or with a well-defined CE stage according to the WHO-IWGE classification, as assessed by abdominal US by an experienced sonographer (EB) (gold standard). When possible, sera were collected from people who had never received treatment for CE or whose treatment ended > 12 months before serum collection. Patients with non-parasitic hepatic cysts were used as controls because non-parasitic cysts represent the most common differential diagnosis of hepatic CE cysts.

### Ethics statement

All patients signed the informed consent for storage and scientific use of the leftover serum at the moment of blood sampling for routine serology. Ethics approval was granted by the Ethics Committee of San Matteo Hospital Foundation, Pavia (approval n. 20150004877).

### Cysts classification

Cysts were classified according to the WHO-IWGE classification. For the analysis, CE cysts were grouped into active (CE1, CE2, CE3a and CE3b) and inactive (CE4 and CE5). Experimental and clinical data prove that CE3b are biologically active (i.e. viable) cysts, while CE3a cysts can be both biologically active or not [16–18]. However, in our analysis, we grouped CE3a cysts with the other active stages as disruption of the integrity of the cyst wall, irrespective of the viability of the cyst, allows parasite antigens to stimulate antibody production. Therefore, it can be speculated that cyst wall integrity is likely a more important condition than the biological viability *per se* to influence serological responses. Patients with small CE1 cysts are often seronegative, although cysts in this stage are unequivocally active [19]; thus this stage should likely be grouped independently in serology analysis. However, not enough samples were present to carry out this sub-analysis. CE4 cysts that reached inactivation spontaneously but recently (or only temporarily inactivated after unsuccessful treatment) should likely also be grouped with “active cysts”, while stably inactive CE4 and CE5 cysts constitute the real “inactive cysts” group [20, 21]. However, this more precise classification at present is not possible in the absence of either long-term follow-up of active cysts without therapy or performing invasive sampling for the assessment of biological activity of cysts, both options burdened by practical and ethical constraints. Therefore, CE4 and CE5 cysts are grouped here in the inactive group. These considerations are at the basis of the choice of cyst grouping used in this study.

### Diagnostic tests

**Fig 1:**
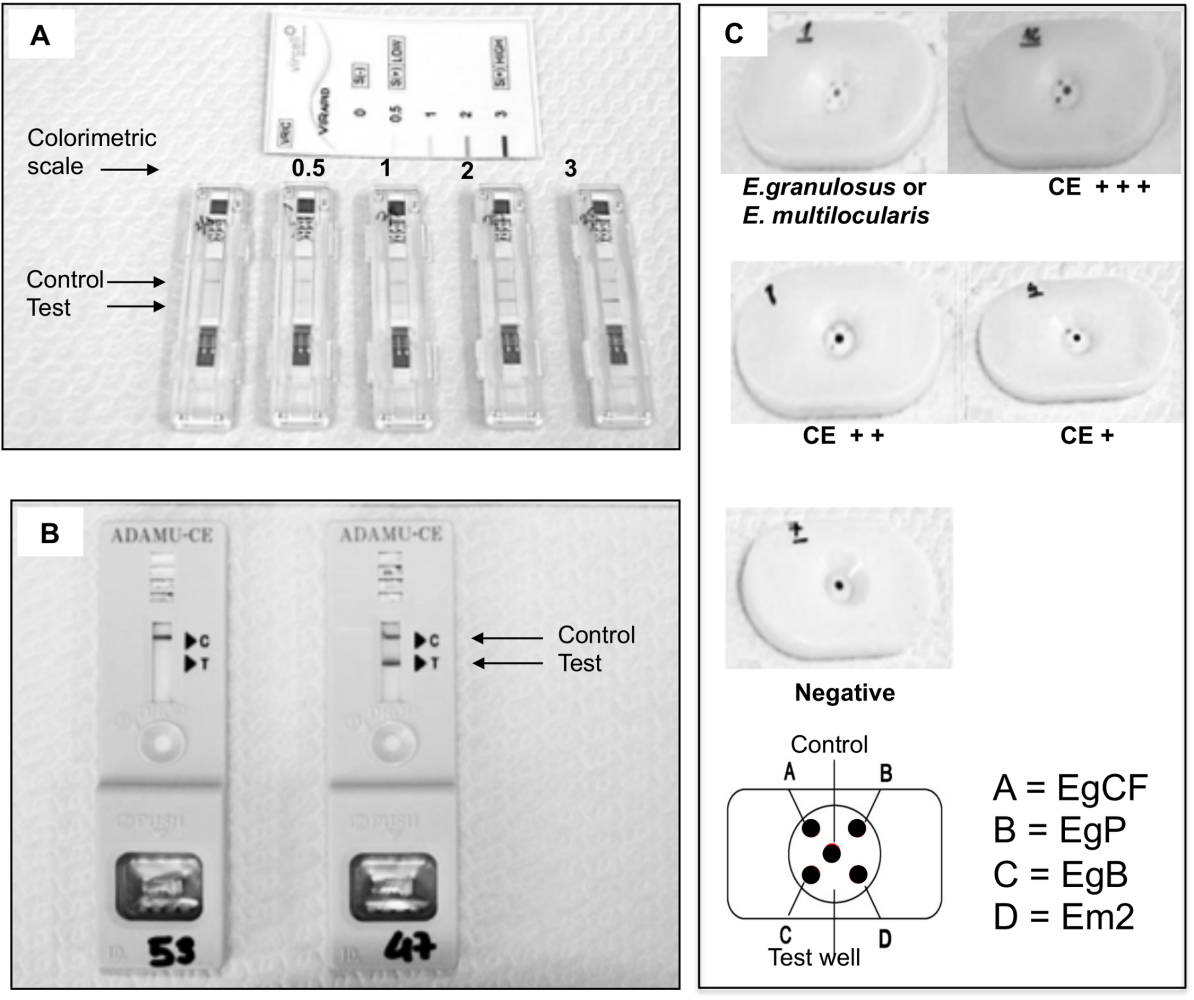
Examples of RDTs results. **(A)** VIRapid HYDATIDOSIS test and its semiquantitative colorimetric scale. **(B)** ADAMU-CE test. **(C)** DIGFA test and its diagnostic and semiquantitative colorimetric interpretation; EgCF = *E*. *granulosus* Cyst Fluid antigen, EgP = *E*. *granulosus* Protoscolex antigen, EgB = *E*. *granulosus* antigen B, Em2 = *E*. *multilocularis* antigen 2.

Selected sera were analyzed using the following three commercially available immunochromatographic rapid diagnostic tests: VIRapid HYDATIDOSIS (based on purified antigen B and antigen 5; Vircell, Salamanca, Spain), Echinococcus Dot Immunogold Filtration Assay (DIGFA, based on purified cyst fluid, protoscolex antigen, antigen B and antigen Em2 of *E*. *multilocularis*; Unibiotest, Wuhan, China), and ADAMU-CE (based on recombinant antigen B; ICST, Saitama, Japan), following the manufacturer’s instructions. The sera were also tested in double with RIDASCREEN Echinococcus IgG ELISA (R-Biopharm, Darmstadt, Germany), routinely used in the parasitology diagnostic laboratory of San Matteo Hospital Foundation, Pavia, Italy, following manufacturer’s instructions. For the ELISA test, Optical Density (OD) results were used to calculate and interpret a Sample Index (SI), as per manufacturer’s instructions. ELISA results were considered positive for SI ≥1.1, negative for SI <0.9, and border line for 0.9 ≤SI<1.1. Borderline results were considered negative for the analysis of results. In this work, “OD” will always refer to Sample Indexes, not to raw OD values; the terminology “OD” was preferred due to more immediate understanding. All tests were performed in parallel in a single session in April 2015. Each test was read by a single operator experienced in laboratory procedures (FT for ELISA and VIRApid, MM for DIGFA, IC for ADAMU-CE). Readers were blind to cyst stage and to results of other tests at the time of reading. Results were recorded as positive or negative and the semiquantitative colorimetric reading of tests was also recorded for VIRapid HYDATIDOSIS and DIGFA tests, as well as OD values for the ELISA test. For DIGFA test, positivity was considered when either “*Echinococcus* spp” or”*E*. *granuolosus* or *E*. *multilocularis*” or “*E*. *granulosus*” indicators were present and the semiquantitative reading was based on the color intensity of the least intense spot. Examples of RDTs results are shown in Fig 1.

### Statistical analysis

The sample size was constrained by the procurement of tests. With a sample of 84 subjects, the study had 80% power for the pairwise comparison of the Area Under the ROC Curve (AUC) of the RDTs, calculated according to the method of Obuchowski [22] and based on the diagnostic performances of the ELISA test, as assessed in a previous work [9]. Difference in AUC was set at 15% and the correlation between two tests at 0.3. Alpha value was set at 0.01 to account for multiple comparisons. The Shapiro-Wilk test was used to test the normal distribution of quantitative variables. When quantitative variables were normally distributed, the results were expressed as the mean value and standard deviation (SD), otherwise median and interquartile range (IQR; 25th -75th percentile) were reported. Qualitative variables were summarized as counts and percentages and differences were analysed with Chi-square test or Fisher exact test, as appropriate. For each test, overall and group-specific (active vs inactive) Sensitivity and Specificity values, as well as AUC, were calculated together with their 95% Confidence Interval (CI). US classification of cysts was considered the gold standard. The performance of the RDTs was compared to those of the ELISA test using McNemar and t-test as appropriate. The semiquantitative reading values of VIRapid HYDATIDOSIS and DIGFA tests were correlated with the ELISA OD values using Spearman’s rank correlation coefficient. Sixteen sera were randomly selected using an electronic random numbers generator and re-analyzed with the three RDTs for assessment of result reproducibility using Cohen’s Kappa coefficient. P<0.05 was considered statistically significant. A Bonferroni-Holm correction was applied for multiple test. All tests were two-sided. The data analysis was been performed with the STATA statistical package (release 13.1, 2014, Stata Corporation, College Station, Texas, USA).

## Results

### Patients and sera characteristics

Eighty-four sera from 84 patients fulfilling inclusion criteria were available for the study. Of these, 59 were patients with single CE cysts of the liver, while 25 had single non-parasitic hepatic cysts. Of the 59 CE patients, 38 had active and 21 inactive cysts, according to the WHO-IWGE classification. Eleven (18.6%) CE patients had received medical treatment with albendazole before sample collection (median 19.4 months before; IQR 10.6–51.6; range 3.1–113.0). The size of the cyst (larger diameter) was not significantly different between active and inactive CE cysts (p = 0.82), while non-parasitic cysts were significantly smaller than CE cysts (p<0.001). Clinical and demographic characteristics of included patients and sera are summarized in Table 1.

**Table 1:** Characteristics of patients and sera included in the analysis.

Group	Gender and age	Active cysts				Inactive cysts		Non-parasitic cysts n = 25
		n = 38				n = 21		
	Gender	CE1 n = 7	CE2 n = 9	CE3a n = 8	CE3b n = 14	CE4 n = 10	CE5 n = 11	
	**Age (y)** median [IQR^§^]	Size (mm)*	Size (mm)	Size (mm)	Size (mm)	Size (mm)	Size (mm)	Size (mm)
		N ABZ (n<1y)^#^	N ABZ (n<1y)	N ABZ (n<1y)	N ABZ (n<1y)	N ABZ (n<1y)	N ABZ (n<1y)	
**CE patients**	26M 33F 53 [39–66]	50 [41–64] 0 (0)	59 [47–60] 1 (0)	59 [49–64] 4 (3)	68 [55–85] 3 (0)	70 [57–76] 3 (0)	57 [39–71] 0 (0)	-
**Controls**	6M 19F 53 [41–71]	-	-	-	-	-	-	40 [24–52]

§ IQR: inter quartile range.

*Size of cysts is expressed as the median of the largest diameter in mm with IQR.

# N ABZ (n<1y): number of subjects having received albendazole before sample collection and number of patients who ended albendazole intake less than 1 year before sample collection (in brackets).

### Sensitivity and specificity of the tests

In one case VIRapid HYDATIDOSIS gave an invalid result (absence of the control band) and was therefore excluded from the analysis. In no case did the DIGFA test give a univocal “*E*. *multilocularis*” result. In 19 (38%) cases (13 out of the 43 [30.23%] CE cysts with positive serology and in 6 out of the 7 [85.71%] non-parasitic cysts with positive serology) DIGFA test failed to individuate *E*. *granulosus*, but provided an “*Echinococcus* spp” result or an “*E*. *granulosus* or *E*. *multilocularis*” result. General test sensitivity and specificity and comparison with the results of the ELISA test are shown in Table 2. The performance of VIRapid HYDATIDOSIS was not statistically different from those of the ELISA test, while those of DIGFA (p = 0.045) and ADAMU-CE (p = 0.074) showed a borderline significant difference.

**Table 2:** Tests sensitivity and specificity. Results are compared with those of the ELISA test. US diagnosis was used as the gold standard. Significant differences are indicated in bold.

Test	N (%) positive in CE group						Overall sensitivity	N (%) positive in Control group	Overall specificity	Chi^2^ and p-value compared to ELISA
	Chi^2^ and p-value compared to ELISA within CE group						(95%CI)	Chi^2^ and p-value compared to ELISA within Control group	(95% CI)	
	Active				Inactive					
	CE1	CE2	CE3a	CE3b	CE4	CE5				
**VIRapid**	43 (74.1%)						74.1% (61.0–84.7)	1 (96%)	96% (79.6–99.9)	χ^2^ = 0.69; p = 0.405
	33 (89.2% [74.6–97.0]) χ^2^ = 0.00; p = 1.000				10 (47.6% [25.7–70.2]) χ^2^ = 1.00; p = 0.317			1 (96% [79.6–99.9]) χ^2^ = 0.00; p = 1.000		-
	4 (66.7%)	8 (88.9%)	8 (100%)	13 (92.9%)	5 (50%)	5 (50%)		-		-
**DIGFA**	43 (72.9%)						72.9% (59.7–83.6)	7 (72%)	72% (50.6–87.9)	χ^2^ = 4.00; p = 0.045
	34 (89.5% [75.2–97.1]) χ^2^ = 0.00; p = 1.000				9 (42.9% [21.8–66.0]) χ^2^ = 0.50; p = 0.479			7 (72% [50.6–87.9]) χ^2^ = 6.00; **p = 0.014**		-
	5 (71.4%)	9 (100%)	8 (100%)	12 (85.7%)	6 (60%)	3 (27.3%)		-		-
**ADAMU-CE**	34 (57.6%)						57.6% (44.1–70.4)	0 (100%)	100% (86.3–100)	χ^2^ = 3.20; p = 0.074
	27 (71.1% [54.1–84.6]) χ^2^ = 5.44; **p = 0.019**				7 (33.3% [14.6–57.0]) χ^2^ = 0.00; p = 1.000			0 (100% [86.3–100]) χ^2^ = 1.00; p = 0.317		-
	4 (57.1%)	7 (77.8%)	7 (87.5%)	9 (64.3%)	3 (30%)	4 (36.4%)		-		-
**ELISA (reference test)**	41 (69.5%)						69.5% (56.1–80.8)	1 (96%)	96% (79.6–99.9)	-
	34 (89.5% [75.2–97.1])				7 (33.3% [14.6–57.0]) -			1 (96% [79.6–99.9]) -		-
	6 (85.7%)	8 (88.9%)	8 (100%)	12 (85.7%)	5 (50%)	2 (18.2%)		-		-

When we analyzed Sensitivity and Specificity of the tests within groups (active, inactive, and non-parasitic), we found that ADAMU-CE was significantly less sensitive in the diagnosis of active cysts (p = 0.019), and DIGFA was significantly less specific when applied on samples from patients with non-parasitic cysts (p = 0.014), compared to ELISA (Table 2). Although a statistical analysis by individual CE stage was not possible due to the limited number of samples, results are indicated in Table 2.

To explore the discrepancies between ELISA and RDTs results, we analyzed the percentage of positive and negative RDTs results of sera from CE patients stratified by ELISA OD groups, set as follows: negative OD < 1.1; low-positive 1.1 ≤ OD ≤ 5.0; high-positive OD > 5.0. The threshold between low-positive and high-positive OD values was set arbitrarily. As shown in Table 3, we found that for all RDTs the percentage of positive results increased passing from negative to low-positive to high-positive ELISA OD groups, with discrepancies between ELISA and RDTs tests being most frequent in the low-positive ELISA OD group.

**Table 3:** Positive and negative results of RDTs stratified by ELISA OD values.

ELISA OD group	RDT test	Positive results	Negative results
Negative	VIRapid	38.89%	61.11%
(OD < 1.1)	DIGFA	33.33%	66.67%
	ADAMU-CE	33.33%	66.67%
Low-positive	VIRapid*	80.00%	16.00%
(1.1 ≤ OD ≤ 5.0)	DIGFA	84.00%	16.00%
	ADAMU-CE	56.00%	44.00%
High-positive	VIRapid	100%	0%
(OD > 5.0)	DIGFA	100%	0%
	ADAMU-CE	87.50%	12.50%

Negative OD < 1.0; low-positive 1.0 ≤ OD ≤ 5.0; high-positive OD > 5.0.

* n = 1 invalid test was excluded from the analysis.

### ROC curves

**Fig 2:**
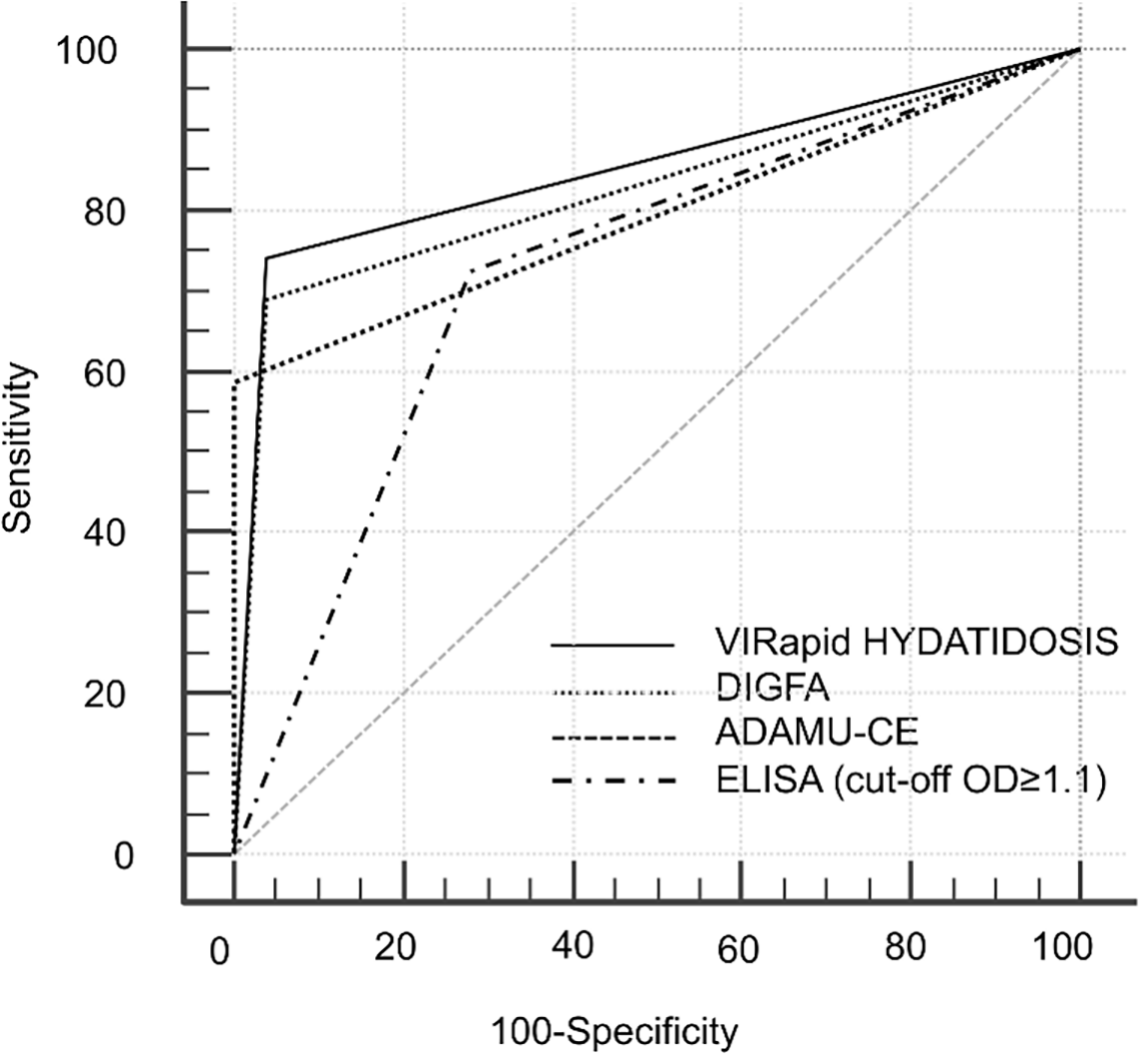
ROC curves of the serology tests.

ROC AUC characteristics and results of comparison between ROC curves are shown in Table 4 and Fig 2. In this analysis a statistically borderline significant difference was seen only between VIRapid HYDATIDOSIS and DIGFA (p = 0.042).

**Table 4:** ROC curves characteristics and results of comparison between tests. AUC = Area Under the Curve [95% CI].

	VIRapid	DIGFA	ADAMU-CE
**VIRapid** AUC 0.851 [0.756–0.919]	-	-	-
**DIGFA** AUC 0.722 [0.616–0.816]	z = 2.028; p = 0.042	-	-
**ADAMU-CE** AUC 0.793 [0.685–0.870]	z = 1.044; p = 0.296	z = 1.022; p = 0.307	-
**ELISA** AUC 0.825 [0.729–0.901]	z = 0.470; p = 0.638	z = 1.710; p = 0.087	z = 0.518; p = 0.604

### Reproducibility

The reproducibility of the RDTs was good (DIGFA, k = 0.71; ADAMU-CE, k = 0.62) to excellent (VIRapid HYDATIDOSIS, k = 1.00).

### Semiquantitative reading

**Fig 3:**
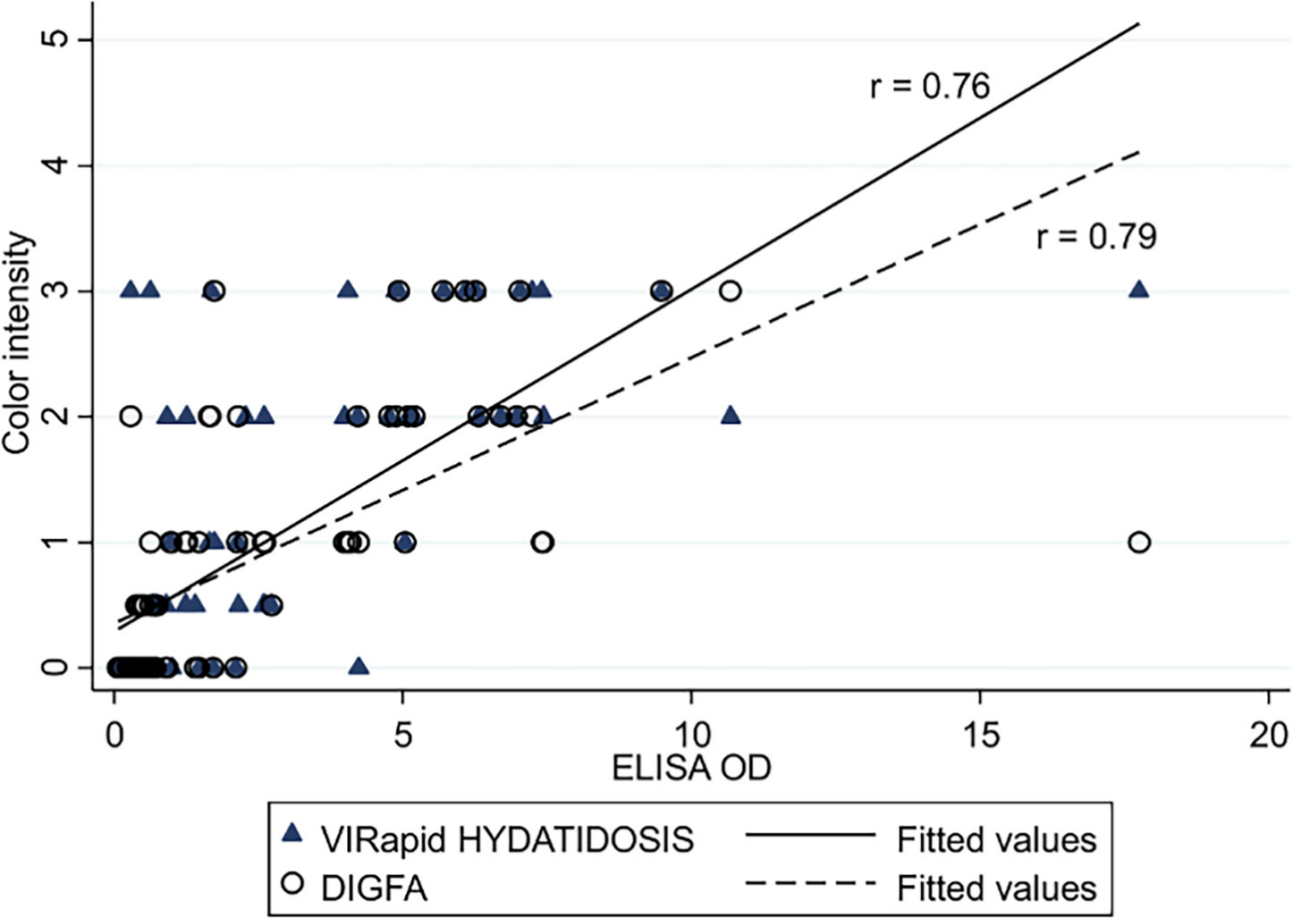
Correlation between ELISA OD values and semiquantitative reading of the VIRapid HYDATIDOSIS and DIGFA tests.

When we examined the correlation between ELISA OD values and the visual semiquantitative reading of band/dots color intensity of VIRapid HYDATIDOSIS and DIGFA, respectively, we found a significant positive correlation in both cases (p < 0.001), as shown in Fig 3.

## Discussion

In rural underserved areas, where CE is most prevalent and health systems are basic and/or difficult to access, the availability of RDTs to help in the differential diagnosis of suggestive US lesions would be very useful. Although several reports described the performance of experimental RDTs in the diagnosis of CE, studies assessing and comparing the diagnostic accuracy of commercially available tests are very scant [10–15].

Feng and colleagues [12], using DIGFA with sera from China, reported a sensitivity of 83.4% for hepatic CE and a specificity of 93.4% when sera came from hospitalized patients. In our centre, the DIGFA test gave clearly inferior results; while our results were comparable with those found by the authors when sera from US screening campaigns were used (Se 71.8% for abdominal CE; Sp 78.1%). Feng and colleagues attributed these differences to the presence, in the field setting, of subjects exposed to the parasite without developing detectable lesions, or the presence of old lesions not accompanied by positive serology. However, sera from hospitalized patients were collected less than 2 years after surgical treatment for CE. Moreover, the authors did not mention the distribution of CE stages in the two patient cohorts. It is therefore likely that the different performance between the two cohorts, and with our results, are at least in part due to the difference in these variables, known to affect serology results. Santivanez and colleagues [23] using a previous form of the ADAMU-CE test on a panel of sera from surgically confirmed CE patients, found a better Se (80% on sera from liver cysts) and same Sp (100% if sera from patients with alveolar echinococcosis were excluded—89.8% if included) compared to our results. In their work, however, they do not provide details of the cyst stages. Therefore, the differences with our results may be at least in part due to differences in these conditions, although different performances between the two “versions” of the kit may not be excluded. Similarly, Tamer and colleagues [14], evaluating the performances of the VIRapid test, reported a better Se (96.8%) and same Sp (96% if sera from patients with other parasitoses were excluded – 87.5% if included) compared to our results, but they did not provide data on cyst characteristics; all CE patients included in their cohort where surgically confirmed, suggesting that predominantly active CE stages were included. Finally, Chen and colleagues [11], using sera from hospital cases, reported that the use of recombinant antigens in the DIGFA test might improve the specificity of the test, but at the expense of sensitivity.

In our centre, the VIRapid HYDATIDOSIS test showed the overall best diagnostic accuracy among the three RDTs, although it did not result statistically significant better than the ELISA test. On the contrary, in comparison with the ELISA test, the ADAMU-CE test was significantly less sensitive in the diagnosis of active cysts, while the DIGFA test was significantly less specific. These results are in line with the literature, reporting overall better sensitivity for tests based on native antigens and better specificity for those based on recombinant antigens [6, 7, 19]. Not surprisingly, all RDTs were as poorly sensitive as the ELISA test in the diagnosis of inactive cysts. These results confirm the limits of serology in the diagnosis of CE and in supporting the differential diagnosis of CE1 and CE4-CE5 cysts from other hepatic lesions. Evidence exist that patients with CE have both common and stage-specific serology profiles, indicating that the development of both infection- and stage-specific immunoassays is possible [24, 25]. Ahn and coworkers [24] showed that antigen 5 seems to be immunoreactive in every stage, as opposed to antigen B, whose proteoforms revealed a reduced antibody capture in CE1, CE4 and CE5 stages. Unfortunately, so far, conventional methods used for antigen discovery such as 2D gel electrophoresis of cyst fluid and immunoblot using sera from infected patients did not allow the identification of stage-specific antigens to be used, alone or in a cocktail, for a more sensitive and stage-specific diagnosis and follow-up of patients. Clearly this should be the focus of high-priority work in the filed.

As mentioned previously, many variables are known to influence CE serology results [9, 19]. In this study, only sera from subjects with a single cyst located in the liver have been included, excluding number and location of the cyst influencing results. Similarly, the size of the cyst was not significantly different between active and inactive cysts; therefore this variable should not have significantly influenced the results. Finally, only 3 out of the 11 CE patients who were treated with albendazole before serum collection ended drug intake less than 12 months prior to sampling. Therefore, the influence of this variable should have only marginally influenced our results given that previous research has shown that treatment that has ended more than 1 year before sampling does not have a significant impact of ELISA test results [9]. The samples size of this study was constrained by the strictness of the inclusion criteria and by the procurement of tests. However, it is pivotal that the first evaluation of diagnostic tests is performed on well-characterized and homogeneous samples. This, unfortunately, is very rarely done, with consequent problems in the interpretation and reliability of the results. The limitation of the number of samples that could be included in this work to comply with this principle was therefore weighted against the quality of baseline data on the evaluation of the RDTs that such an approach could provide. In this work, we included sera from patients with non-parasitic cysts as controls because non-parasitic cysts represent the most common differential diagnosis of hepatic CE cysts. Surely further work should thoroughly evaluate the specificity of the tests with sera from patients with other parasitoses, in particular alveolar echinococcosis. However, it must be stressed that serology for CE should be performed only after lesions compatible with echinococcosis are found by imaging, to increase the pre-test probability of the presence of infection. Indeed, due to the low prevalence of infection (and consequent very low Positive Predictive Value of any test), the generally low specificity of serodiagnostic tests (especially an issue in areas where contact with the parasite without cyst development and number of other diseases affecting the population may be significant), and the very low sensitivity of serodiagnosis in extra-hepatic CE (limiting the use of serology to diagnose CE in organs not explorable by ultrasound), the value of serological screenings is limited and should be conducted only after careful evaluation of the scientific question such studies want to answer.

To conclude, our results show that RDTs have overall comparable performance to the routine ELISA test in the diagnosis of hepatic CE in well-defined stages, although significant differences in diagnostic accuracy exist among them. These results support their use in resource-poor settings to complement ultrasound diagnosis of CE in doubtful cases. However, all tests are poorly sensitive in the presence of inactive and CE1 cysts, which are cyst stages that may pose considerable problems of differential diagnosis. Furthermore, further studies are warranted to explore the performance of RDTs in the follow-up of CE patients, often extremely difficult to perform with regular US examinations in endemic areas. VIRapid HYDATIDOSIS appeared to perform best among the examined kits and deserves further testing with a larger cohort including other control groups (e.g. with other parasitoses) and sera from patients with extra-hepatic CE cysts and with CE cysts of different parasite genotypes. The test also deserves further evaluation in the field and with the use of whole blood from fingerprick sampling. Finally, benefit studies on the use of RDTs, and serology tests in general, are lacking in the field of CE and deserve future efforts.

## Supporting Information

S1 Checklist STARD checklist. (PDF)

S1 Flowchart Study flowchart. (PDF)

S1 Table Original data. (XLS)
