# Supplementary figures and images for: Comparison of the Diagnostic Accuracy of Three Rapid Tests for the Serodiagnosis of Hepatic Cystic Echinococcosis in Humans
Source: PLoS Negl Trop Dis. 2016 Feb 12;10(2):e0004444. doi: 10.1371/journal.pntd.0004444 (PMC4752287; doi:10.1371/journal.pntd.0004444)

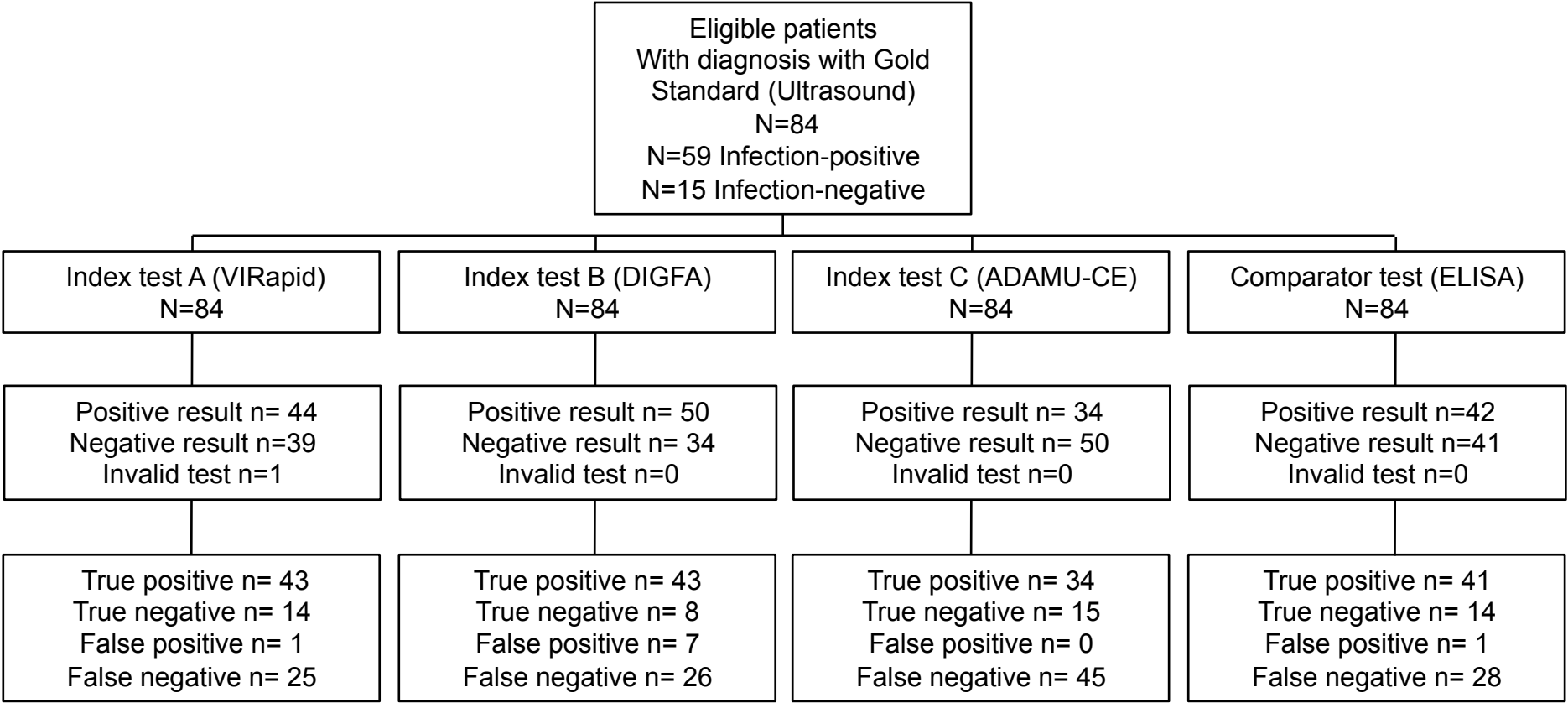

Supplement: S1 Flowchart — (PDF) [file pntd.0004444.s002.pdf]
